# Amphetamine manipulates monoamine oxidase-A level and behavior using theranostic aptamers of transcription factors AP-1/NF-kB

**DOI:** 10.1186/s12929-016-0239-2

**Published:** 2016-02-03

**Authors:** Christina H. Liu, Jiaqian Ren, Philip K. Liu

**Affiliations:** Athinoula A. Martinos Center for Biomedical Imaging, Department of Radiology, Massachusetts General Hospital, Charlestown, MA 02129 USA; Present address: NIH, 6707 Democracy Blvd, Suite 200, Bethesda, MD 20892 USA

**Keywords:** Acute stress, Addiction, Bipolar, Oxidative stress, Parkinson dementia, Protein-targeted delivery, Theranostics

## Abstract

**Background:**

Monoamine oxidase (MAO) enzymes play a critical role in controlling the catabolism of monoamine neurotransmitters and biogenic trace amines and behavior in humans. However, the mechanisms that regulate MAO are unclear. Several transcription factor proteins are proposed to modulate the transcription of *MAO* gene, but evidence supporting these hypotheses is controversial. We aimed to investigate the mechanism of gene transcription regulator proteins on amphetamine-induced behavior. We applied aptamers containing a DNA binding sequence, as well as a random sequence (without target) to study the modulation of amphetamine-induced MAO levels and hyperactivity in living mice.

**Methods:**

We pretreated in adult male C57black6 mice (Taconic Farm, Germantown, NY) (n ≥ 3 litters at a time), 2 to 3 months of age (23 ± 2 gm body weight) with double-stranded (ds) DNA aptamers with sequence specific to activator protein-1 (5ECdsAP1), nuclear factor-kappa beta (5ECdsNF-kB), special protein-1 (5ECdsSP-1) or cyclicAMP responsive element binding (5ECdsCreB) protein binding regions, 5ECdsRan [a random sequence without target], single-stranded AP-1 (5ECssAP-1) (8 nmol DNA per kg) or saline (5 μl, intracerebroventricular [icv] injection) control before amphetamine administration (4 mg/kg, i.p.). We then measured and analyzed locomotor activities and the level of MAO-A and MAO-B activity.

**Results:**

In the pathological condition of amphetamine exposure, we showed here that pretreatment with 5ECdsAP1 and 5ECdsNF-kB reversed the decrease of MAO-A activity (*p* < 0.05, *t* test), but not activity of the B isomer (MAO-B), in the ventral tegmental area (VTA) and substantia nigra (SN) of C57black6 mice. The change in MAO-A level coincided with a reversed amphetamine-induced restless behavior of mice. Pretreatments with saline, 5ECdsCreB, 5ECdsSP-1, 5ECdsRan or 5ECssAP-1 had no effect.

**Conclusion:**

Our data lead us to conclude that elevation of AP-1 or NF-kB indirectly decreases MAO-A protein levels which, in turn, diminishes MAO-A ability in the VTA of the mesolimbic dopaminergic pathway that has been implicated in cells under stress especially in the SN and VTA. This study has implications for design for the treatment of drug exposure and perhaps Parkinson’s dementia.

## Background

Amphetamine-type stimulants (Ecstasy, methamphetamine, 3-4-methylenedioxymethamphetamine (MDMA or MOLLY]) are second only to marijuana as the top drugs of abuse worldwide (2011 Global ATS assessment, a United Nations report). According to the National Drug Abuse Warning Network, the emergency room visits were more than double from 2004 to 2009 due to taking MDMA alone or in combination with pharmaceuticals, alcohol or both. MDMA is a Schedule I drug and its use has the potential for abuse and impairment in learning [1], aggressive behavior as well as symptoms such as those seen in schizophrenia and depression [2, 3]. The mechanism of action is not totally understood, except that amphetamine and its analogs induce elevation of gene transcription regulator proteins AP-1 and NF-kB. Both transcription factors are known to response to oxidative stress, inflammatory and apoptotic signals [4], but reduce striatal astrocyte population with a concomitant elevation of astrocytes in the subventricular zone, a clear indication of brain damage [5]. The relationship between elevations of oxidative biomarkers and abnormal psycho-physiological behavior after amphetamine is less clear. Recently, it was shown that amphetamine could reduce MAO-A among others proteins [6], and importantly this was found in HIV-1 transgenic rats which normal metabolism of the brain may have been altered [7].

MAO catalyzes the oxidative deamination of endogenous monoamines in the human body [8]. There are two isoforms of MAO (MAO-A and MAO-B), localized in outer mitochondrial membrane; they are distributed throughout the nervous system as well as other regions of the body including the digestive and circulatory systems. MAO-A deficiency resulting from mutation in the *MAO-A* gene can cause a characteristic set of symptoms (i.e., mild mental retardation, impulsive antisocial behavior, and mood and panic disorders) collectively referred to as Brunner syndrome [9]. Mutations in the mouse *MAO* gene confer the same phenotype [10]; therefore, the mouse is an ideal animal model for preclinical experiments involving MAO-A manipulation.

Interestingly, elevated transcription factor proteins AP-1 and NF-kB and microglial activation are also known associations in ischemia-reperfusion injury, Parkinson disorder model, and human immunodeficiency viral infection. Variation in *MAO-A* expression has been associated with polymorphisms in the promoter region [11, 12], which was also shown to contain at least one consensus sequence for AP-1 and SP-1 binding [13–16]. To better understand how these two TF proteins may influence MAO-A activity, we have taken an alternative approach using a double-stranded (ds) DNA aptamer with consensus sequences for these TF proteins. We have demonstrated the specific and sensitive binding of the dsAP1 and dsNF-kB aptamers to AP-1 and NF-kB proteins, respectively [5], including a null binding to the AP-1 aptamer in mice with mutant AP-1 proteins [17]. MAO-A is found in the cytoplasm of dopaminergic neurons in the SN, pars compacta, hypothalamus and VTA of the midbrain. Because the mesolimbic neurons of the reward pathway have axonal projections originating from the VTA, we examined MAO-A antigen in the VTA region. Here, we correlate the effect of dsAP-1/NF-kB aptamers on motor activities with MAO-A expression in the VTA of live mice exposed to amphetamine.

## Methods

### Animals and housing

All of the procedures used in this study were approved by the Massachusetts General Hospital Subcommittee on Research Animal Care, the institutional animal welfare committee, in accordance with the Public Health Service Guide for the Care and Use of Laboratory Animals. Adult male C57black6 mice (Taconic Farm, Germantown, NY) (n ≥ 3 litters at a time), 2 to 3 months of age (23 ± 2 gm BW), were kept in cages on sawdust bedding, in a room with controlled light cycles (12 h light/12 h dark), where they had free access to water and were fed standard lab chow. Mice were trained, operated on, and tested in a randomized manner, with a blinded observer performing the behavioral testing.

### Short dsDNA for AP1 binding protein

Double-stranded DNA containing the consensus sequence (denoted by uppercase letters) for AP1 protein (5’-fluorecein isothiocyanate [FITC]-tccggcTGACTCAtcaagcg-3’ and 3’-aggccgACTGAGTagttcgc-biotin-5’) were modified by phosphorothioation, a process by which sulfur replaces non-bridging oxygen on the phosphate linkages of three, four, or five nucleotides (lowercase letters) from both ends (end caps, EC). Additional 5ECdsDNAs for transcription factors include SP1 (5ECdsSP-1, 5’-ctcgcCCCGCCccgatcgaa-biotin and 3’-gagcgGGGCGGggctagctt); nuclear factor kappa beta (5ECdsNF-κβ, 5’-agttgaGGGGACTTTCCcaggc-biotin and 3’ tcaactCCCCTGAAAGGgtccg) and cyclic AMP response element binding protein (5ECdsCREB, 5’-ctctcTGACGTCAggcaat-biotin and 3’-gagagACTGCTGTccgtta). When single-stranded DNA was used as a control, the oligoDNA was fully modified by phosphorothioation.

Both strands of the transcription factor binding sequences were mixed at room temperature, heated at 65 °C for five minutes, and slowly cooled on a thermocycler (1 degree drop per minute) to 20 °C, at which temperature they were maintained for 30 min. Short dsDNAs were stored in aliquots of 0.05 ml (100 μM) at −20 °C. One hour before use, one aliquot was thawed to room temperature.

### Delivery of 5ECdsDNA aptamers

For uptake studies we anesthetized the mice with pure O_2_ plus 2 % halothane at a flow rate of 800 ml/min, and delivered the dsDNA aptamers (8 nmol DNA per kg, *n* = 4 each) by icv injection [18, 19], an accepted route of delivery for administering contrast agents to rodents and nonhuman primates [20–22]. We delivered 8 nmol DNA per kg (icv) for TF protein knockdown studies [23]. Three hours after DNA delivery we administered either saline (0.1 ml, i.p.) or amphetamine (4 mg per kg, i.p.). The uptake of various 5EDdsDNA aptamer was performed and validated as described previously; all photographs were obtained with the same exposure time, using a Himatsu CCD camera on an Olympus microscope (Optical Analysis Corp, NH) [5].

### Behavioral testing after amphetamine

All mice were preconditioned for at least 48 h in the testing cage, which was equipped with a locomotion detector [21]. We delivered dsDNA or a control (icv) three hours before we administered amphetamine [23]. After amphetamine delivery the mice were immediately placed in their home cages, where we measured their locomotion for 60 min.

### Animal handling

In order to minimize the effect of stress animals may experience by being in different environments before and during amphetamine stimulation, each mouse was housed in the same cage throughout the experiments, which included one week of habituation to a new environment. Three days before amphetamine pre-treatment, we habituated all animals by administering a daily injection of saline solution (0.25 ml) and assessing behavior as baseline on each day. Each home cage was placed directly in the automatic recording system before animals received their amphetamine injection. We divided animals into two groups to receive either amphetamine or saline (vehicle) pretreatment for the subsequent days. The mice were never placed in a new cage for behavioral assessment as described [21].

## MAO protein expression

### Immunohistochemistry

We delivered saline, dsAP1, or dsRan to mice by icv injection (2 μl), and amphetamine described above; tissue samples were obtained from mice 90 min after amphetamine. We measured MAO-A expression using rabbit polyclonal antibodies against MAO-A(1:500, Novus Biologicals, Littleton, CO, NBP1-19796) using a procedure previously published [19].

### Western blot of MAO protein expression

Tissue samples (5 mg) of mouse VTA were removed after 90 or 180 min using a biopsy punch (Miltex Inc York, PA); the samples were then snap frozen in liquid nitrogen and homogenized using an ultrasonicator (Sonic Dismenbrator Model 100, Fisher Scientific, Pittsburgh, PA) in 100 μl of cold radioimmunoprecipitation assay lysis buffer with a protease inhibitor cocktail (Sigma-Aldrich, St. Louis, MO). The homogenate was then centrifuged for 10 min at 12,000 g at 4 °C. The protein concentration was determined by the Bradford method (BioRad Protein Assay, Hercules, CA), using a reference curve from bovine serum albumin. Protein samples (10 μg each group) mixed from four mice in each group, along with a molecular weight marker ladder (10 KD ladder, Thermo Scientific, Rockford, IL) were separated on a 20 % tris-glycine PAGE (Invitrogen) and transferred to a PVDF membrane using a semidry electrophoretic blotter (BioRad). The primary rabbit polyclonal antibodies against MAO-A or MAO-B were diluted at 1:1000 (Novus NBP-59569 for MAO-B). Anti-actin rabbit polyclonal antibodies (1:1000 dilutions, Abcam, ab1801, Cambridge, MA) were added with IgG against MAO for control detection. Chemiluminescent detection was performed with a Western Breeze chemiluminescent kit (Invitrogen, Grand Island, NY) that includes anti-rabbit secondary antibodies, and the signal was captured on an X-ray film (Kodak BioMax XAR film, St. Louis MO). Quantitative densitometry of protein bands was performed using an image-processing program (FluorChem Q, Alpha Innotech, San Jose, CA). In addition to the VTA proteins, housekeeping β-actin proteins were used as normalizing factors, and as an indicator of the quality of antibody binding. All assays were repeated at least twice to ensure reproducibility.

### Statistical analysis

Once we had obtained the first data set, we calculated the number of animals needed in each group to achieve 95 % power for a *p* value of 0.01 [21, 24]. We computed the mean and standard error of the mean (SEM) from the average values in each group of animals, and compared the statistical significance of these values using a *t* test (one tail, type II or equal variant, GraphPad Prism IV, GraphPad Software, Inc., San Diego, CA). A *p* value of less than 0.05 was statistically significant.

## Results

### Amphetamine reduces MAO-A antigen level

**Fig. 1:**
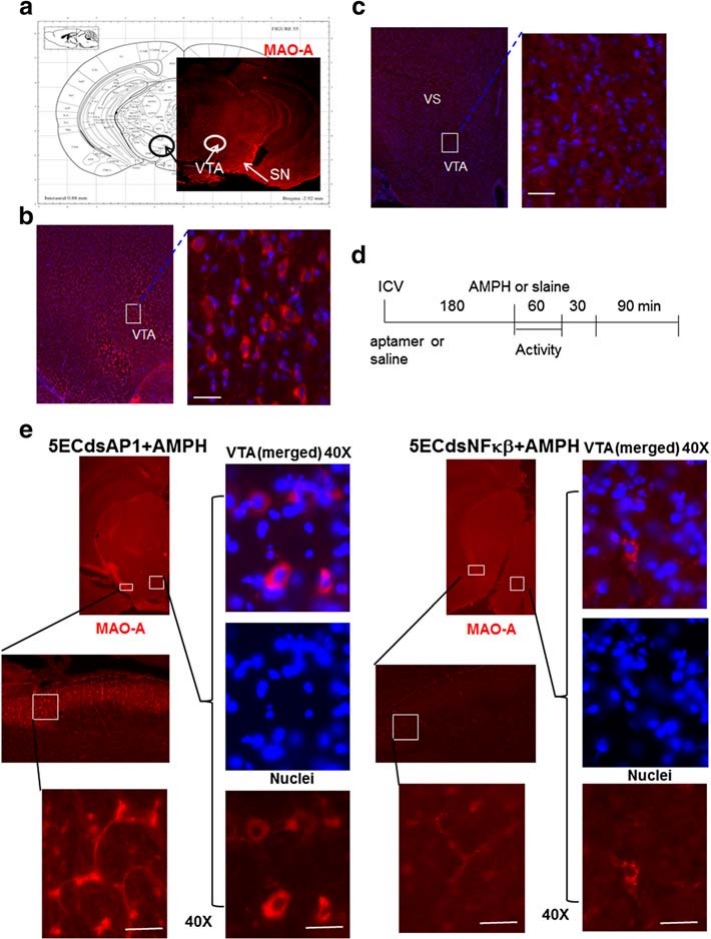
The expression of MAO-A antigen in the CNS (no injection, panel **a**) in mice after saline (100 μl, i.p., panel **b**) or amphetamine (4 mg/kg, s.c., panel **c**) as shown in the protocol (panel **d**). Pretreatment of AP-1 or NF-kB Aptamer reverses AMPH-induced MAO-A deficiency (panel **e**). Mice pretreated with 5ECdsAP-1 or 5ECdsNF-kB aptamer before amphetamine (AMPH) show a reversal of MAO-A protein expression. MAO-A protein is present in the cytoplasm of the soma in the VTA (ventral tegmental area and axons in the SN (substantia nigra) and VS (ventral striatum) of the mesolimbic pathway. Immunohistochemistry results suggested that 5ECdsAP-1 might have a different temporal effect from 5ECdsNF-kB did on the reversal of MAO-A antigen level

We found the presence of MAO-A in the cytoplasm of dopaminergic neurons, which in naïve mice are present chiefly in the SN, pars compacta, hypothalamus and VTA of the midbrain (circles, Fig. 1a). In normal mouse brains without amphetamine MAO-A is distributed within the soma and axons of the neurons in the VTA (boxes, Fig. 1b). Amphetamine treatment reduced MAO-A antigen in the mesolimbic neurons of the SN, ventral striatum and VTA (boxes, Fig. 1c).

To investigate the mechanism by which amphetamine reduces MAO-A, we applied aptamers with consensus sequences for TF proteins as well as one aptamer with random (Ran) sequence, which has no intracellular target. We applied 5ECdsAP1 or 5ECdsNF-kB (n ≥ 2 each group) to mice according to the protocol outlined in Fig. 1d. Mice with 5ECdsAP1 or 5ECdsNF-kB aptamer pretreatment showed a reversal of amphetamine-induced MAO-A reduction in the soma of the VTA neurons as well as in the axons of neurons in the SN (Fig. 1e).

### Modulation of transcription factor AP-1 protein on MAO-A activity

**Fig. 2:**
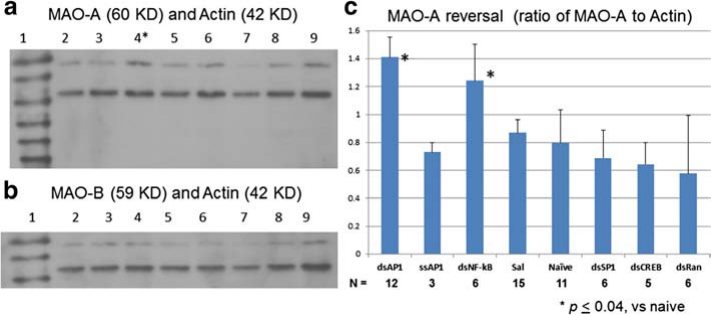
MAO-A and MAO-B levels after amphetamine. We pretreated mice with 5ECdsAP1 aptamer before amphetamine application according to Fig. 1d; we collected tissue samples (*n* = 4 per group) from the VTA at 90 (60 + 30 min Fig. 1d) and 180 (60 + 30 + 90 Fig. 1d) minutes after amphetamine. We obtained protein for Western blot quantitation of MAO-A (panel **a**) or MAO-B (panel **b**) level (upper molecular fragments) using Actin (lower molecular fragments) as a reference shown in lanes 2–4 (90 min samples) or lane 5–6 (180 min). Total protein (10 μg) was used for all lanes except lanes 7 & 9. The blot was stripped and used for MAO-B after MOA-A. Lane 1: molecular size marker of 10 KD ladder; lane 2: 5ECdsAP1 (4 nmol/kg, icv, half dose); lanes 3 & 5: saline (2 μl, icv); lanes 4 & 6: 5ECdsAP1 (8 nmol/kg, icv, full dose); lanes 7, 8, 9 are controls of naive mice (no aptamer or amphetamine (with increasing amount of protein: 5, 10 and 20 μg, respectively. Because we observed no change in MAO-B level (Panel **b**), we determined 90 min post amphetamine is the optimal time to collect VTA samples (panel **a**, lane 4) for quantitative comparison of the reversal of MAO-A level in the VTA tissue from mice treated with 5EC aptamers of dsAP1, ssAP1, dsNF-kB, saline (Sal), nothing (naïve), dsSP1, dsCREB, and dsRan (panel **c**). Aptamer 5ECdsAP1 elevated the MAO-A by 60–100 % (*t* test, shown as bar graphs in panel **c**). N = number of mice used in the test

We quantitatively measured MAO-A level by Western blot, using Actin as a control. MAO-A antigen levels, as measured in VTA samples (n ≥ 3 each lane, three measurements or 12 – 15 mice) collected 90 min after amphetamine (Fig. 2a), were elevated with 5ECdsAP1 pretreatment (lane 4, Fig. 2a). A half dose of 5ECdsAP1 (4 nmol/kg) did not reverse amphetamine’s effect on MAO-A (lane 2). The results that we acquired 180 min after amphetamine exposure showed only slight elevation, with no significant difference in MAO-A levels associated with either 5ECdsAP1 treatment (lane 6) or naïve mice (lane 8, Fig. 2a). MAO-B expression was unchanged in the same sets of samples (Fig. 2b). Pretreatment with 5ECdsNF-kB reversed MAO-A activity (Fig. 2c). Moreover, we observed no significant change in MAO-A level following pretreatment with either control (5ECdsRan or saline), single-stranded aptamer (ssAP1) 5ECdsSP1 or 5ECdsCREB aptamers (Fig. 2c); these results thus establish that the reversal of MAO-A reduction is significant and specific to 5ECdsAP1. Furthermore, RT-PCR and TaqMan analysis showed no significant change in MAO-A mRNA before and after 5ECdsAP1 and amphetamine administration (*p* = 0.05, *t* test, two *n* = 4 samples from each group). Together these data reveal that amphetamine exposure reduces MAO-A protein, which in turn produces an antagonist effect on amphetamine-induced acute stress behaviors. We conclude that the modulation of MAO-A level in the VTA is inversely related to AP-1 transcription factor expression.

### 5ECdsAP1 ameliorates amphetamine-induced motor activity

**Fig. 3:**
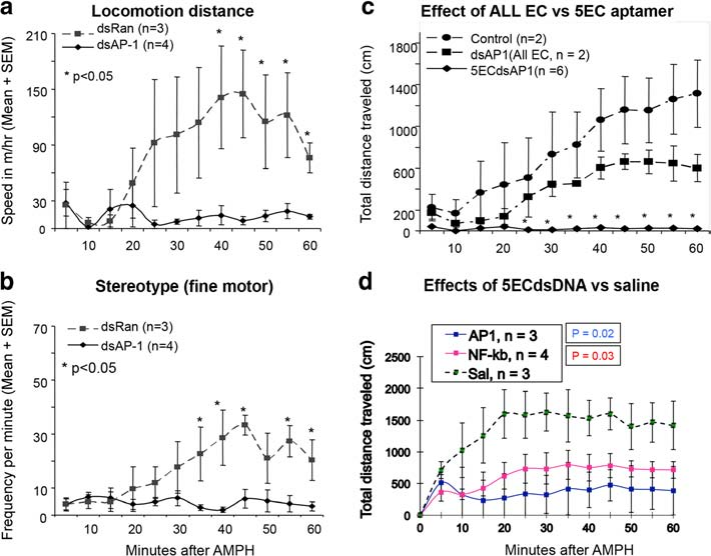
Amphetamine-induced hyperlocomotor activity in the control group [pretreatment with 5ECdsRan (panels **a** & **b**) or saline (panels **c** & **d**) before amphetamine] is reversed to near normal levels when mice were pretreated with 5ECdsAP1 (panels **a** – **d**) or 5ECdsNF-kB (panels **c** & **d**). Other control group not shown in this graph is pretreatment with ssAP-1 (fully phosphorothioate-modified). Fine motor movement (front paw movement without changing body position) is shows as frequency per minute (panel **b**). Distance traveled is given at speed (m per hr (panel **a**) or in centimeters at each interval of 5 min (panels **c** & **d**, which was calculated by the frequency of ambulation (locomotion between adjacent beams of light of 5 cm in one minute) x 5 cm per minute x 5 min per recoding interval. Typically, normal mice without treatment exhibited no more than 30 ambulation frequency per min within 15 min after handling (baseline). No significance was found between the control (saline) and dsAP-1 of Full EC (panel **c**)

We recorded and measured locomotor activities using an automatic recording system in a controlled environment [21]. We then compared the effect on motor activity by measuring the distance the mice traveled when given a minimum dose of 8 nmol/kg of 5ECdsAP1, 5ECdsNF-kB, 5ECdsRan or saline. We observed that pretreatment with 5ECdsAP1 or 5ECdsNF-kB aptamer significantly suppressed an amphetamine-induced increase in locomotor activity (*p* < 0.05). We did not observe in this suppression of amphetamine-induced hyperactivity in control mice that received saline or 5ECdsRan (not shown) before amphetamine (Fig. 3). Moreover, 5ECdsAP1 with complete phosphorothioate linkage (dsAP1, All EC), which is known to have sticky properties for all proteins, partially attenuated the hyperactivity suppression effect. A half dose of 5ECdsAP1 (i.e., 4 nmol/kg, icv) had no significant effect on hyperactivity suppression, nor did single-stranded sODN-AP1 (ssAP-1) at full dose (not shown).

## Discussion

Amphetamine exposure induces elevations in immediate early genes and their products; however, the relationship of such changes in gene expression to amphetamine induced restless activity is not clear. Here, we have applied nucleic acid aptamers to to AP-1 and NF-kB and examined how MAO-A and MAO-B proteins modulate amphetamine-induced hyperactivity. Our results show that AP-1 and NF-kB activities are responsible for the reduction in the level of MAO-A, but not MAO-B, by a post transcription pathway; the effect of MAO-A change is inversely related to hyperactivity after amphetamine. A reduced level of MAO-A correlates with hyperactive behavior; our study suggests that MAO-A induced aggressive behavior can be modulated via epigenetic means. We show that a reduced MAO-A level (Fig. 1) without changing MAO-B level (Fig. 2) caused abnormal behavior (Fig. 3); phenomenon observed also in humans and in rats [7]. Although this observation is different from consensus understanding that MAO-B activities metabolize dopamine in normal condition of human brains [25], we believe there was no conflicts because the modulation of MAO-A we studied is pathological condition during amphetamine exposure where we observed a resistance of MAO-B to amphetamine. Our study also supports results from a study showed that NF-kB protein knockdown using antisense strategy reduced amphetamine-mediate feeding suppression [26]. Indeed, hyperthermia, oxidative stress, neuro-toxicity and striatal neuroglia loss are severe complications associate with amphetamine exposure [27]; AP-1 and/or NF-kB TF proteins can initiate these biological processes. However, most studies have suggested that the hyperactivity observed in association with amphetamine results from altered neurotransmitter activities in dopaminergic neurons of the mesolimbic pathway, which to date has been regarded as resulting from a dopamine transporter-mediated elevation in extracellular monoamine [28]. Here, we show using aptamer competing with AP-1/NF-kB TF protein binding that MAO-A level is reversed in the VTA leading to suppression of amphetamine-induced behavioral abnormality in the animal.

Our study demonstrates the specificity of the aptamer for MAO-A, as 5ECdsAP1 had no effect on MAO-B. This specificity supports regulation of different pathways by these two isomers [29, 30]. Because our aptamer effectively competes with in vivo TF proteins, which are hetero- and/or homo-dimeric forms of at least two gene families, our aptamer provides a direct approach to gene knockdown of proteins and acts as an antagonist indirectly modulating the level of MAO-A protein.

Our observation that specific reduction of MAO-A protein happened without significant change in MAO-A mRNA in the VTA after amphetamine exposure, suggests that MAO transcription activity is not affected directly by TF-mediated regulation on MAO genes, especially involving the SP-1 transcription factor [31]. We propose that the reduction in MAO-A protein may be a result of catabolic action to exogenous amphetamine detoxification, most likely as an inflammatory reaction related to AP-1/NF-kB TF proteins. Moreover, the absence of significant MAO-A mRNA elevation after amphetamine suggest that the reduction in MAO-A does not produce a feedback signal fast enough to increase MAO-A transcription, or the signal is blocked by a suppressor. The suppressor hypothesis supports the result of histone deacetylation on the promoter of c-fos gene [32]. The mechanism proposed here may also explain the null effect that cocaine elicits on hyperactivity observed in other studies in a double-transgenic mouse strain that produces no active AP-1 TF protein [17].

Both AP-1 and NF-kB TF proteins are known to increase after amphetamine exposure. Similar phenomenon has been described in animal models of ischemia reperfusion brain injury, major mood disorder and/or bipolar disorder, Parkinson disease [33, 34] and in human populations infected with sexually transmitted infections [4, 35, 36]. Acute stress, characterized by anxiety (insomnia, irritability, poor concentration, motor restlessness), among other human conditions, can be experimentally induced in animals [37, 38]. Given that reduced MAO-A protein levels are associated with acute stress in humans [39], we envision that our 5ECdsAP1/5ECdsNF-kB aptamer may be applied, in theory, to correct abnormal MAO-A expression observed during acute stress.

OligoDNA antisense to Fos gene families reduced TF protein by knockdown of respective mRNA [40, 41]. We have shown that aptamer 5ECdsAP-1 or 5ECdsNF-kB targets AP-1 or NF-kB TF protein on a gel shift assay, respectively [5]. Specific targeting in living brains mediate MRI of intracellular TF protein at low dose and reduce amphetamine-mediated MAO-A enzyme degradation and stress activities at high dose. The latter event keeps a normal level of MAO-A in the mesolimbic dopaminergic neurons supports that MAO-A level is inversely proportional to hyperactivity. The data supports the association of MAO-A activity and normal behavior [10]. Therefore, our study confirms that targeting MR-CA using aptamers can have a potential for theranostic application [21].

We did not observe a complete reversal of amphetamine-induced hyperactivity in response to dsAP1 with total phosphorothioation (Fig. 2). We attribute this effect to nonspecific binding of the aptamer to other proteins, which hence dilutes the effective binding on the target AP-1 protein. A number of studies by other investigators have used aptamers to target different TF proteins [42–45]. In addition to 5ECdsCreb aptamer, 5ECdsSP-1 failed to modulate MAO-A level because SP-1 binding site is absent from the human promoter of the *MAO-A* gene but can be found at the promoter of the human *MAO-B* gene [31]. We have clarified the mechanism by which the aptamer mitigates amphetamine-related hyperactivity via MAO-A attenuation; our method, therefore, provides an additional means to evaluate the mechanism and the internal trafficking of these TF proteins in vivo.

## Conclusion

Because aptamer can be labeled with iron oxide nanoparticles, gold, or fluorescent dyes our approach may be applied for multimodal imaging using MRI (in vivo) and microscopy (ex vivo). The results are consistent with the report that 5ECdsAP1 or 5ECdsNF-kB can specifically guide the delivery of DNA or contrast agents to the cells that contain the target of unique intracellular TF proteins. Furthermore, an intracerebroventricular injection, which directly bypasses the blood–brain barrier, is clinically accepted for delivery of therapeutic agents, validating the translational potential of our approach. We anticipate that this technique will have potential for real-time and longitudinal research, in preclinical as well as for clinical applications.

## Abbreviations

FITC: fluorescein isothiocyanate
Icv: intracerebroventricular
MAO-A: monoamine oxidase-A
NAc: nucleus accumbens
SN: substantia nigra
TF: transcription factor
VTA: ventral tegmental area
